# Inappropriate and potentially avoidable emergency department visits of Swiss nursing home residents and their resource use: a retrospective chart-review

**DOI:** 10.1186/s12877-022-03308-9

**Published:** 2022-08-11

**Authors:** Franziska Zúñiga, Katharina Gaertner, Sabine K. Weber-Schuh, Barbara Löw, Michael Simon, Martin Müller

**Affiliations:** 1 Nursing Science (INS), Department Public Health (DPH), Faculty of Medicine, University of Basel, Basel, Switzerland; 2 Institute of Integrative Medicine, Witten/Herdecke University, Witten, Germany; 3 Department of Emergency Medicine, Inselspital, Bern University Hospital, Bern, Switzerland; 4 Department of Practice Development in Nursing, Solothurner Spitaler AG, Solothurn, Switzerland; 5 GP practice, Praxis Weissenbühl, Bern, Switzerland

**Keywords:** Nursing homes, Long-term care, Emergency departments, Hospitalization, Inappropriate, Avoidable, Resource consumption, Quality management

## Abstract

**Background:**

Emergency department (ED) visits for nursing home residents lead to higher morbidity and mortality. Therefore, inappropriate visits (for conditions treatable elsewhere) or potentially avoidable visits (those avoidable through adequate chronic care management) must be minimized. This study aimed to investigate factors and resource consumption patterns associated with inappropriate and potentially avoidable visits in a Swiss tertiary hospital.

**Methods:**

This is a single-center retrospective chart review in an urban Swiss university hospital ED. A consecutive sample of 1276 visits by nursing home residents (≥ 65 years old), recorded between January 1, 2015 and December 31, 2017 (three calendar years) were included. Case characteristics were extracted from ED electronic documentation. Appropriateness was assessed via a structured Appropriateness Evaluation Protocol; potentially avoidable visits—measured as ambulatory-care sensitive conditions (ACSCs)—were analyzed separately. Inter-group differences concerning ED resource use were tested respectively with chi-square or Wilcoxon rank sum tests. To identify predictors of inappropriate or potentially-avoidable visits, we used multivariable logistic regression analysis.

**Results:**

Six percent of visits were rated as inappropriate: they had lower triage levels (OR 0.55 [95%-CI 0.33-0.92], *p=*0.024) and, compared to ambulance calls, they had higher odds of initiation via either patient-initiated walk-in (OR 3.42 [95%-CI 1.79-6.55], *p≤*0.001) or GP referrals (OR 2.13 [95%-CI 1.16-3.90], *p=*0.015). For inappropriate visits, overall ED resource use was significantly lower (median 568 vs. 1403 tax points, *p≤*0.001). Of all visits included, 29% were due to (often potentially-avoidable) ACSCs. In those cases, compared to ambulance initiation, odds of being potentially-avoidable were considerably lower for walk-in patients (OR 0.46 [95%-CI 0.27-0.77], *p=*0.004) but higher for GP referrals (OR 1.40 [95%-CI 1.00-1.94], *p=*0.048). Nurse work (93 tax points vs. 64, *p≤*0.001) and laboratory resource use (334 tax points vs. 214, *p≤*0.001) were higher for potentially-avoidable ED visits.

**Conclusions:**

We revealed substantial differences between the investigated groups. While nearly one third of ED visits from nursing homes were potentially avoidable, inappropriate visits were lower in numbers and not resource-intensive. Further research is required to differentiate potentially avoidable visits from inappropriate ones and to determine these findings’ public health implications.

**Supplementary Information:**

The online version contains supplementary material available at 10.1186/s12877-022-03308-9.

## Background

Emergency department (ED) visits are stressors for nursing home residents and are linked to adverse outcomes such as deliriums or falls [1–3]. Nursing home residents are more frequently frail and have higher levels of multimorbidity than their community-dwelling peers [4]. Their most common reasons for ED admissions are falls, infections, altered mental status, and respiratory or circulatory problems [5–8]. Overall, visiting rates of nursing home residents to the ED are rising [3, 9, 10] and, as higher age is related to increased length of stay at the ED, these visits contribute to ED overcrowding [11].

More importantly, approximately 1% to 5% of admitted nursing home residents die in the ED [1, 2]. One suggested reason for high ED visit rates from nursing homes is a lack of advance care planning (ACP) [8]. Other reasons include lack of access to primary care services in the nursing home, insufficiently trained staff, as well as understaffing, lack of access to specialists’ medical advice and poor interprofessional collaboration [8, 12]. Overall, depending on the definition and measurement method, 5% to 55% of ED visits are considered potentially inappropriate or avoidable [2, 8].

While the terms *inappropriate* and *avoidable* are often used interchangeably regarding visits, we differentiate between them. Potentially inappropriate visits are for “condition [s] which could have been treated in the nursing home or somewhere else than in the ED” [13], i.e., residents might have visited their general practitioner (GP) during office hours to assess their symptoms, for example, rather than immediately going to the ED; and potentially avoidable visits are for “condition [s] which could have been avoided to be treated in the ED, if adequate chronic care management was provided at the nursing home” [13]. An example would be an adequate management of congestive heart disease with regular weighing of the resident and corresponding adaptation of diuretics when needed to avoid an exacerbation of pulmonary symptoms, which would need to be treated in a hospital. This differentiation helps to clarify the underlying mechanisms of nursing home ED admissions that are likely reduceable via timely nursing home interventions. I.e., potentially inappropriate visits are based on missing or false assessments of clinical situations, for which further observation and a scheduled visit with a GP would be an appropriate course of action; potentially avoidable visits indicate problems in chronic care management, including daily symptom assessment and timely reaction to changes. Omitting such measures can lead to exacerbations that demand immediate interventions.

From an ED perspective, any visit for a non-emergency (somatic or psychiatric) situation, for which treatment could have been provided by a GP, or dealing with residents receiving palliative care or non-hospitalization orders in their files might be considered potentially inappropriate [12, 14]. Such ED visits can be rated by expert panels or via appropriateness assessment protocols [12, 15–17]—for example, gauging the severity of patient symptoms and whether any indicated clinical interventions could have been provided elsewhere.

Whereas gauging a visit’s appropriacy is guided by protocols or done by expert ratings, potentially avoidable nursing home resident visits are often measured with ambulatory-care sensitive conditions (ACSCs) [18–21]. Frequently, conditions such as urinary tract infections, congestive heart failure, chronic obstructive pulmonary disease (COPD) or pneumonia are described as ACSCs [18, 19]. That is, if changes are detected early and the necessary treatments provided, they are considered manageable in outpatient settings. Although this rating is not conclusive and a multi-layered approach is needed to assess each transfer individually, ACSCs provide a first impression of commonly avoidable reasons for ED visits. Only recently have studies begun to differentiate systematically between potentially inappropriate and potentially avoidable ED visits [12, 13].

ED visits not only include a risk of harm to the resident; they also increase healthcare costs by increasing the use of ED resources regarding both working time of ED employees (mainly physicians and nurses) and diagnostics and materials. To date, few studies have addressed ED resource use [22]. However, one New York State study found that 23% of costs for nursing home residents’ hospitalizations were attributable to ACSCs [23], i.e., were very likely avoidable. A Turkish study found that the mean cost per nursing home resident ED visit was 337 USD, not including laboratory analyses or radiology [24]. And our literature search returned no publications addressing nursing home residents’ ED resource use where the authors differentiated between potentially inappropriate and potentially avoidable visits.

Therefore, this study’s primary aim was to investigate rates of potentially avoidable ED visits (due to ACSCs) and those judged as potentially inappropriate in a Swiss tertiary hospital. Secondary aims were to assess the corresponding case characteristics, including patient comorbidities, to compare ED resource use for both types of ED visits, and to describe factors associated with both potentially inappropriate and potentially avoidable ED visits.

## Methods

### Design, setting and sample

**Fig. 1:**
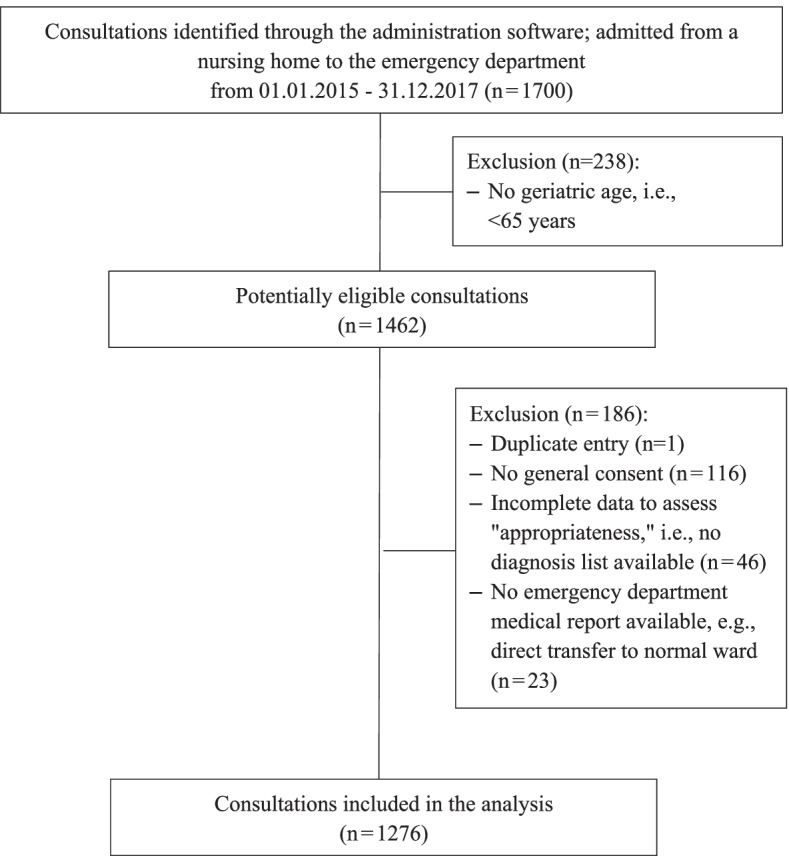
Flowchart

This single-center observational retrospective chart-review analyses used data from the ED of an urban Swiss university hospital with a catchment area of roughly two million people and about 45’000 ED consultations per year [25]. We included electronic health records from January 1, 2015 to December 31, 2017 (three calendar years). Our two inclusion criteria were specification as “referral from nursing home” in the ED’s E-Care record (E-Care, ED 2.1.3.0, Turnhout, Belgium); and an age of 65 years or older. As the expected rates of potentially avoidable ED visits and those judged as potentially inappropriate were unclear, we assumed a rate of 10%, with confidence intervals of 0.082-0.121 for n=1’000. In the given timeframe, a consecutive sample of 1’462 records were eligible with patients of at least 65 years of age. We excluded 186 cases (12.7%) mainly due to either lack of consent or incomplete data (s. Figure 1), analyzing a consecutive sample of 1276 nursing home residents.

### Data collection

Data were retrieved by medical analyses of the ED’s electronic health records. All data for analysis were pseudonymized, meaning only the medical analysts and ED personnel could connect a patient’s case number with their identity. Files of patients who objected to the use of their data in health research were excluded from the analysis.

### Variables and measurements

To assess potentially avoidable ED visits, we identified ACSCs based on papers by Walker & Teare [18] and Walsh & Wiener [19], who tailored their ACSC definition to the nursing home context. We chose an overlap of 13 diagnoses presented in both papers: congestive heart failure, hypertension, hypotension, diabetes, constipation, pressure ulcer, angina, COPD/pneumonia, dehydration, urinary tract infections, seizure, gastroenteritis, and cellulitis. Screening the text field in the ED documentation where diagnoses were formulated as free text allowed us to identify patients with ACSC diagnoses as the reason for their admission.

Inappropriate ED visits were assessed with Finn et al.’s adapted Appropriateness Evaluation Protocol [17]. That assessment consists of a dichotomized 10-item list to determine the absence or presence of ten characteristics within patient consultations: i) hospitalization needed; ii) a history of trauma with suspected fracture; iii) radiology examination needed; iv) plaster application needed; v) suspicion of a cerebral event; vi) difficult indwelling urethral catheter insertion; vii) percutaneous endoscopic gastrostomy tube insertion; viii) requirement of intravenous antibiotics; ix) suitable observations unavailable; and x) procedure not performable in a nursing home. The items were assessed sequentially. As soon as one item was confirmed in the case record, the visit was rated as "appropriate." If all items were absent, the visit was rated as "inappropriate" (see Supplement 1).

Demographic data (age and gender) were used to describe the studied population. To describe patterns of presentation, three variables were assessed: time (0 am to 12 pm) of arrival in the ED; day of arrival (Monday to Sunday); and type of admission (i.e., ambulance, walk-in, physician’s referral) were assessed. Additionally, four outcome parameters were extracted from the administrative database (OpenText Suite for SAP® Solutions, OpenText Corp., Waterloo, Canada): length of stay (LOS) in the ED (hours); LOS in the hospital (days); Intensive care unit (ICU) admission (yes/no); and in-hospital death (yes/no).

Furthermore, each patient’s triage rating and Charlson Comorbidity Index (CCI) score were analyzed. The triage rating was measured with the Swiss triage scale (range: 1–4; 1 = acute emergency requiring immediate treatment; 2 = treatment required within twenty minutes; 3 = treatment required within 120 minutes; and 4 = a non-urgent situation) [26]. The CCI was developed to predict ten-year mortality due to comorbid conditions [27]. Fourteen chronic conditions are rated as either absent or present, with three—diabetes, malignancy and liver disease—weighted differently depending on their severity. Scores range from 0 to 33, with a score of 7 or more indicating a 0% probability of ten-year-survival [28]. Following Halfon & Eggli’s methods [28], we used the CCI to assess each patient’s comorbidity status [29].

Lastly, ED resources (physician and nurse work, laboratory and radiology resources) were assessed using tax points (TP). As required by Swiss health care law, all staff members documented their performed procedures individually for every consultation, using procedural codes from the TARMED Suisse catalogue (TARMED Suisse, TARMED, 01.06.2012). The TARMED codes are measured in TPs, a medical currency in Switzerland. One TP is roughly equal to 1 USD, depending, among other points, with exact values depending on the hospital and region. Based on the study hospital’s TP value, we calculated total ED costs. Additionally, we reported physician time resources used, differentiating between time spent with patients and time devoted to medical reports and other administrative tasks.

### Data analysis

Data were analyzed descriptively, using medians, interquartile ranges, and percentages as appropriate. Data were checked for completeness; if basic data such as hospitalization were missing, the case was removed.

All visits were coded for ACSCs and appropriateness by two of the co-authors (BL, SS). In unclear cases, two other authors were consulted (MM, FZ). To assess interrater reliability, we used Cohen’s kappa and Gwet’s AC1. With low prevalence rates, Cohen’s kappa is relatively inaccurate, while Gwet’s AC1 is reliable across the full range of input [30, 31]. For the adapted AEP, reviewer agreement was 93.1%. Cohen’s kappa was moderate (unweighted kappa = 0.46), and Gwet’s AC1 was very good (Gwet’s AC1= 0.92). Regarding ACSCs, reviewer agreement was 80.8%. Cohen’s kappa was moderate (unweighted kappa = 0.52), and Gwet’s AC1 good (Gwet’s AC1= 0.68). Based on the calculated interrater reliability, the raters compared all their results and discussed unclear cases.

To assess differences between ACSC-based and other visits, and between inappropriate and appropriate visits, we performed the Wilcoxon sum rank test or Chi-Square test respectively for interval-scaled or categorical variables. To describe relationships between patient characteristics and consultation characteristics with avoidable visits, we built an exploratory logistic regression model. For a second model with inappropriate visits as the outcome, comorbidities were added. To ensure comparability between the predictor variables, we either dichotomized or categorized them. As a first step, all variables from the descriptive data analysis with a *p-*value <0.2 were added to the model. Next, non-significant variables were removed. Goodness of fit was tested using the Hosmer and Lemeshow test. The strength of each association was presented as an odds ratio accompanied by its 95% confidence interval (CI). A *p-*value of <0.05 was considered significant. Data analyses were conducted using IBM SPSS Statistics Version 27.

## Results

One thousand two hundred and seventy-six nursing home residents at least 65 years of age were included in the analyses. Over the observation period, the frequency of ED visits rose from 367 nursing home residents in 2015 to 416 in 2016 and 493 in 2017. The involved residents were a median age of 84 years old; their most common comorbidities were chronic kidney disease (36%), dementia (32%), cerebrovascular disease (24%) and diabetes (22%). More than half were admitted during daytime; the days of arrival were distributed nearly equally across the week. Two-thirds were admitted by ambulance (see Table 1).

**Table 1:** Patient and consultation characteristics and comorbidities in potentially inappropriate and avoidable ED visits

	Overall (***n=***1,276)		Potentially inappropriate (***n=***77)		Appropriate (***n=***1,199)		***p-***value	Potentially avoidable (ACSC) (***n=***373)		Non-avoidable (non-ACSC) (***n=***903)		***p-***value
Age, [Med (IQR)]	84.0	(78-89)	84.0	(77-88)	84.0	(79-89)	0.284	83.0	(77-88)	85.0	(79-90)	<0.001
Sex, [n (%)]												
Female	793	(62.1)	50	(64.9)	743	(62.0)		229	(61.4)	564	(62.5)	
Male	483	(37.9)	27	(35.1)	456	(38.0)	0.603	144	(38.6)	339	(37.5)	0.721
**Consultation characteristics**												
Day of the week, [n (%)]												
Sunday	171	(13.4)	10	(13.0)	161	(13.4)		42	(11.3)	129	(14.3)	
Monday	181	(14.2)	10	(13.0)	171	(14.3)		62	(16.6)	119	(13.2)	
Tuesday	180	(14.1)	13	(16.9)	167	(13.9)		51	(13.7)	129	(14.3)	
Wednesday	206	(16.1)	10	(13.0)	196	(16.3)		56	(15.0)	150	(16.6)	
Thursday	192	(15.0)	8	(10.4)	184	(15.3)		60	(16.1)	132	(14.6)	
Friday	176	(13.8)	17	(22.1)	159	(13.3)		52	(13.9)	124	(13.7)	
Saturday	170	(13.3)	9	(11.7)	161	(13.4)	0.373	50	(13.4)	120	(13.3)	0.558
Visit time, [n (%)]												
7:00 - 15:59	789	(61.8)	53	(68.8)	736	(61.4)		230	(61.7)	559	(61.9)	
16:00 - 23:59	310	(24.3)	18	(23.4)	292	(24.4)		85	(22.8)	225	(24.9)	
0:00 - 6:59	177	(13.9)	6	(7.8)	171	(14.3)	0.239	58	(15.5)	119	(13.2)	0.457
Night visit (18:00 – 6:59), [n (%)]	355	(27.8)	16	(20.8)	339	(28.3)	0.155	105	(28.2)	250	(27.7)	0.866
Type of admission, [n (%)]												
Ambulance	855	(67.0)	36	(48.6)	819	(73.5)		254	(72.4)	601	(71.8)	
General practitioner	191	(15.0)	19	(25.7)	172	(15.4)		72	(20.5)	119	(14.2)	
Walk-in	112	(8.8)	16	(21.6)	96	(8.6)		18	(5.1)	94	(11.2)	
Other	30	(2.4)	3	(4.1)	27	(2.4)	<0.001	7	(2.0)	23	(2.7)	0.001
Triage, [Med (IQR)]	2.0	(2-3)	3.0	(2-3)	2.0	(2-3)	<0.001	2.0	(2-3)	2.0	(2-3)	0.739
Triage, [n (%)]												
Life-threatening	250	(19.8)	1	(1.3)	249	(21.0)		63	(17.1)	187	(21.0)	
Urgent conditions	458	(36.3)	26	(33.8)	432	(36.5)		146	(39.6)	312	(35.0)	
Semi-urgent conditions	532	(42.2)	45	(58.4)	487	(41.1)		157	(42.5)	375	(42.0)	
Non urgent conditions	21	(1.7)	5	(6.5)	16	(1.4)	<0.001	3	(0.8)	18	(2.0)	0.122
**Comorbidity**												
Charlson Comorbidity Index, [Med (IQR)]	6.0	(5-8)	6.0	(4-8)	6.0	(5-8)	0.041	7	(5-9)	6	(4-8)	<0.001
Congestive heart failure, [n (%)]	150	(11.8)	5	(6.5)	145	(12.1)	0.139	76	(20.4)	74	(8.2)	<0.001
Past myocardial infarction, [n (%)]	127	(10.0)	6	(7.8)	121	(10.1)	0.514	54	(14.5)	73	(8.1)	0.001
Chronic kidney disease, [n (%)]	455	(35.7)	22	(28.6)	433	(36.1)	0.180	164	(44.0)	291	(32.2)	<0.001
Diabetes, [n (%)]	280	(21.9)	17	(22.1)	263	(21.9)	0.977	96	(25.7)	184	(20.4)	0.035
Liver disease, [n (%)]	81	(6.3)	4	(5.2)	77	(6.4)	0.669	34	(9.1)	47	(5.2)	0.009
Chronic obstructive lung disease, [n (%)]	135	(10.6)	11	(14.3)	124	(10.3)	0.275	58	(15.5)	77	(8.5)	<0.001
Dementia, [n (%)]	410	(32.1)	22	(28.6)	388	(32.4)	0.490	141	(37.8)	269	(29.8)	0.005
Cerebrovascular disease, [n (%)]	301	(23.6)	10	(13.0)	291	(24.3)	0.024	65	(17.4)	236	(26.1)	0.001
Hemi-/Paraplegia, [n (%)]	212	(16.6)	6	(7.8)	206	(17.2)	0.032	46	(12.3)	166	(18.4)	0.008
Peripheral artery occlusive disease, [n (%)]	98	(7.7)	10	(13.0)	88	(7.3)	0.071	31	(8.3)	67	(7.4)	0.587
Connective tissue disease, [n (%)]	7	(0.5)	3	(3.9)	4	(0.3)	<0.001	4	(1.1)	3	(0.3)	0.104
Peptic ulcer, [n (%)]	41	(3.2)	3	(3.9)	38	(3.2)	0.726	14	(3.8)	27	(3.0)	0.482
Malignancy, [n (%)]	267	(20.9)	10	(13.0)	257	(21.4)	0.077	87	(23.3)	180	(19.9)	0.176
**Outcome**												
Hospitalization, [n (%)]	878	(68.8)	0	(0.0)	878	(73.2)	<0.001	311	(83.4)	567	(62.8)	<0.001
ICU transmission, [n (%)]	194	(15.2)	0	(0.0)	194	(16.2)	<0.001	58	(15.5)	136	(15.1)	0.825
In-hospital death, [n (%)]	83	(6.5)	0	(0.0)	83	(6.9)	0.017	25	(6.7)	58	(6.4)	0.854

*Abbreviations*: *ACSC* Ambulatory-care Sensitive Condition, *ED* Emergency Department, *ICU* Intermediate Care Unit, *IQR* Interquartile Range, *LOS* Length of stay; *Med* Median

Notes: the CCI’s max. value in this study was 15, even though the original score ranges from 0 to 33

### Potentially inappropriate visits

**Fig. 2:**
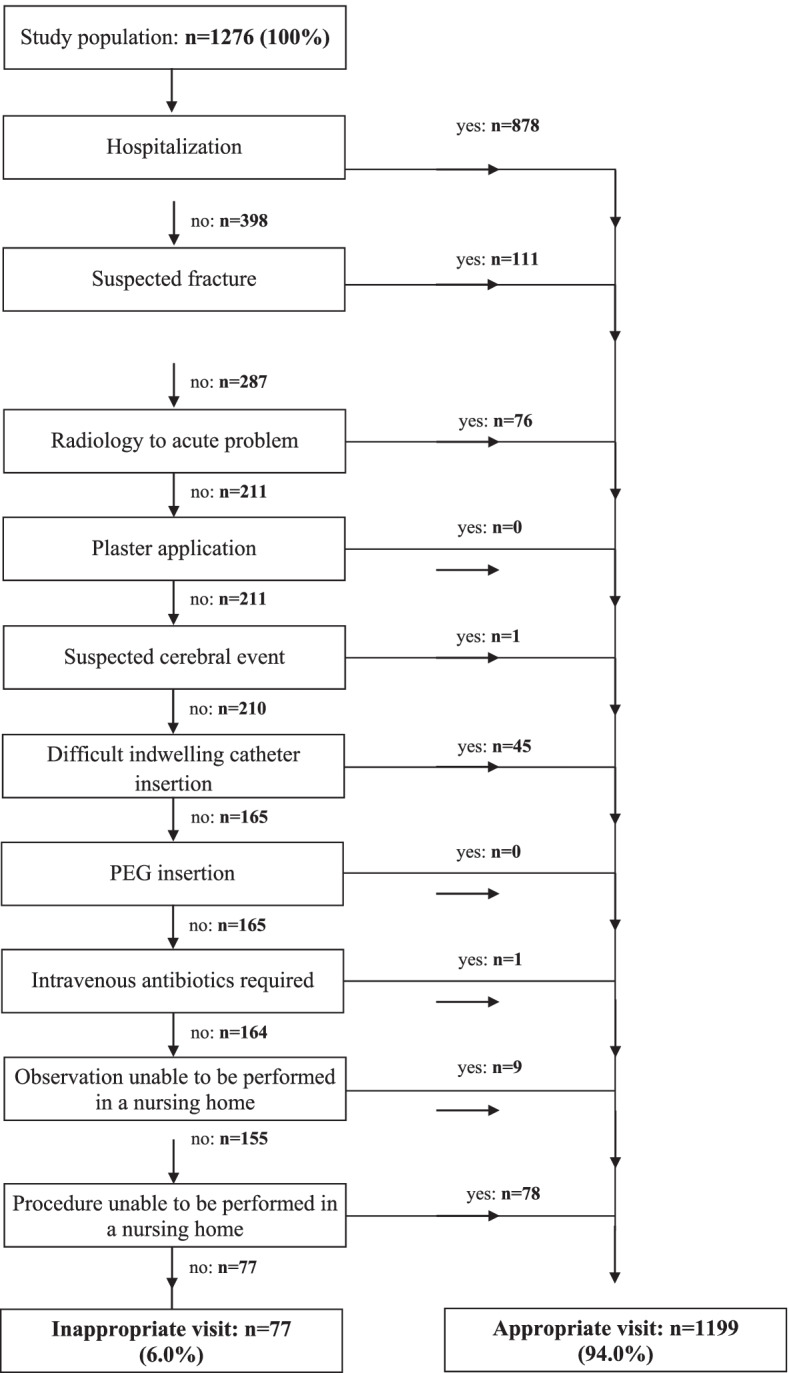
Inappropriate admissions

Figure 2 shows the characteristics of the patients whose records were assessed for potential inappropriateness: overall, 6.0% (*n=*77) of the analyzed cases were assessed as including potentially inappropriate visits. Roughly two-thirds of the sample (*n=*878; 68.8%) had to be hospitalized. Of the remaining 398, many had a history of trauma with suspected fracture (*n=*111; 8.7%), needed radiological examinations (*n=*76; 6.0%), were referred for procedures that could not be performed in the nursing home (*n=*78; 6.1%), or were affected by difficult indwelling catheter insertion (*n=*45; 3.5%).

Compared to residents whose visits proved appropriate, those with potentially inappropriate visits were more often admitted as walk-in patients (21.6% vs. 8.6%) or referred by their GPs (25.7% vs. 15.4%). Those with potentially inappropriate visits were also more often (correctly) triaged as non-urgent (6.5% vs. 1.4%). Regarding specific co-morbidities, potentially inappropriate visits were less likely to be for cerebrovascular diseases (13.0% vs. 24.3%, *p=*0.024) or hemi-/paraplegia (7.8% vs. 17.2%, *p=*0.032) and more likely to be related to connective tissue diseases such as systemic lupus erythematosus or scleroderma (respectively 3.9% vs. 0.3%, *p<*0.001). Following this study’s definition of potentially inappropriate visits, none of the people whose visits were potentially inappropriate were hospitalized.

### Potentially avoidable visits

Of all visits, 373 (29.2 %) were for ACSCs. The most commonly rated were COPD/pneumonia (98 cases, 26.3%), urinary tract infection (84 cases, 22.5%), and congestive heart failure (50 cases, 13.4%). Occasionally, hypertension (40 cases, 10.7%), cellulitis (32 cases, 8.6%), and seizures (29 cases, 7.8%) were rated, followed first by dehydration (20 cases, 5.4%), then by diabetes and gastroenteritis (13 cases each, 3.5%).

Of the 373 residents whose visits were considered potentially avoidable, most (72.4%) were admitted by ambulance. Triage ratings for avoidable visits were similar to all visits; but residents admitted with ACSCs had higher Charlson Comorbidity Index scores (7 vs. 6, *p<*0.001) and had a higher incidence of hospitalization 83.4% vs. 62.8%, *p<*0.001).

### ED Resource use

While potentially inappropriate visits by nursing home residents used considerable ED resources, their median total ED resource cost was significantly lower than for appropriate visits. This was true both for tax points (median 568 vs. 1403 tax points; *p<*0.001) and for Swiss francs (Median CHF 805 vs. CHF 1567, *p<*0.001). This difference is reflected in significantly lower use of physician work, nurse work, as well as laboratory and radiology resources (TP) for potentially inappropriate visits when compared to appropriate ones (Table 2). ED physicians’ time expenditures (both with patients and writing medical reports) did not differ significantly between the groups (*p=*0.052, resp. 0.056).

**Table 2:** Comparison of ED resource use for inappropriate and avoidable visits

	Total (***n=***1276)		Potentially inappropriate (***n=***77)		Appropriate (***n=***1199)		***P-***value	Potentially avoidable (ACSC) (***n=***373)		Non-avoidable (non-ACSC) (***n=***903)		***P-***value
	Med	IQR	Med	IQR	Med	IQR		Med	IQR	Med	IQR	
**ED resource use (TP)**												
Total resources [TP]	1327	(715-1849)	568	(226-828)	1403	(795-1889)	<0.001	1317	(895-1817)	1334	(606-1871)	0.136
Resource subgroups:												
Physician work [TP]	506	(297-668)	391	(155-518)	506	(314-681)	<0.001	519	(359-678)	506	(265-666)	0.189
Nurse work [TP]	87	(35-93)	35	(0-58)	93	(35-93)	<0.001	93	(58-112)	64	(35-93)	<0.001
Laboratory resources [TP]	251	(92-390)	81	(0-181)	267	(117-408)	<0.001	334	(203-528)	214	(62-351)	<0.001
Radiology resources [TP]	318	(58-838)	0	(0-64)	345	(58-887)	<0.001	202	(58-652)	400	(0-917)	0.038
**ED resource use (time)**												
Physician patient time [min]	60	(35-85)	60	(25-70)	60	(40-85)	0.052	60	(45-85)	60	(30-80)	0.013
Physician medical report time [min]	20	(11-20)	20	(11-20)	20	(11-20)	0.056	20	(11-22)	20	(11-20)	0.009
Physician admin time [min]	55	(30-80)	35	(15-50)	60	(30-80)	<0.001	60	(35-80)	55	(25-75)	0.030
**Administrative outcomes**												
LOS hospital (days)	2.1	(0.2-6.7)	0.2	(0.1-0.2)	2.8	(0.2-6.8)	<0.001	5.0	(0.9-8.9)	1.1	(0.2-5.1)	<0.001
LOS ED (hour)	5.0	(3.2-7.1)	3.8	(2.5-6.0)	5.0	(3.3-7.2)	0.008	5.4	(4.0-7.4)	4.7	(2.9-7.0)	<0.001
Total ED costs per patient [Swiss Francs]*	1515	(785-2118)	805	(472-1133)	1567	(837-2161)	<0.001	1538	(1021-2037)	1502	(674-2177)	0.663

* 1 Swiss Franc ≈ 1.003 USD; The values are presented as median (interquartile range) if not declared otherwise

*Abbreviations*: *ACSC* Ambulatory-care Sensitive Condition, *ED* Emergency Department, *IQR* Interquartile Range, *LOS* Length of Stay, *Med* Median, *TP* Tax Points

In contrast, compared to non-avoidable visits, those that were potentially avoidable necessitated more physician-patient time, admin time and medical report time, as well as nurse work and laboratory resources. As potentially avoidable visits required fewer radiology resources, their total ED resource use and total ED costs did not differ from those of non-avoidable ones. Still, compared to nursing home residents visiting for non-avoidable reasons, those visiting for potentially avoidable reasons stayed significantly longer both in the ED (5.4 vs 4.7 h, *p<*0.001) and in hospital (5.0 vs. 1.1 days, p <0.001).

### Predictors of potentially inappropriate and avoidable visits

The multivariable logistic regression model to predict potentially inappropriate visits is shown in Table 3. For potentially inappropriate visits, the odds were lower for acute triage classification or malignancy and higher for connective tissue disease, e.g., lupus. Additionally, compared to visits initiated via ambulance calls, those following GP referrals had 2.1-fold higher odds (OR 2.13, 95%-CI 1.16-3.90) and patient-initiated walk-ins 3.4-fold higher odds (OR 3.42, 95%-CI 1.79-6.55) of being potentially inappropriate. The Hosmer and Lemeshow test indicated a moderate fit (*p=*0.061).

**Table 3:** Logistic regression models for inappropriate and avoidable visits

Variable	Potentially inappropriate ED visits (***n=***1’174 included in modelling)			Avoidable ED visits (***n=***1’188 included in modelling)		
	Odds Ratio	(95% CI)	***p-***value	Odds Ratio	(95% CI)	***p-***value
Age				0.98	(0.96-0.99)	0.005
Triage score: life-threatening/urgent	0.55	(0.33-0.92)	0.024			
Type of admission (reference: ambulance)						
General practitioner	2.13	(1.16-3.90)	0.015	1.40	(1.00-1.94)	0.048
Walk-in	3.42	(1.79-6.55)	<0.001	0.46	(0.27-0.77)	0.004
Other	2.06	(0.58-7.27)	0.262	0.73	(0.31-1.73)	0.471
No Hospitalization				2.51	(1.81-3.47)	<0.001
Charlson Comorbidity Index				1.10	(1.05-1.16)	<0.001
No connective tissue disease	11.68	(2.46-55.48)	0.002			
No malignancy	0.48	(0.23-0.99)	0.048			
Constant	0.07		<0.001	1.55		0.538
	Chi-Square	Df		Chi-Square	Df	
Hosmer and Lemeshow Test	12.06	6	0.061	7.40	8	0.496

*Abbreviations*: *CI* Confidence Interval, *ED* Emergency Department

Our model associated potentially avoidable ED visits with 2.5-fold higher odds of hospitalization than non-avoidable ones (OR 2.51, 95%-CI 1.81-3.47). Similarly, potentially avoidable visits had 1.4-fold higher odds of originating from GP referrals (OR 1.40, 95%-CI 1.00-1.94) but 46% lower odds of patient-initiated walk-in (OR 0.46, 95%-CI 0.27-0.77) than of ambulance calls. Details of these results are presented in Table 3.

## Discussion

The aim of this study was to investigate a) rates and patterns of potentially inappropriate and avoidable ED visits by nursing home patients, b) ED resource use arising from such visits, and c) variables that affect their occurrence. Overall, we found that, while only 6% of ED visits were potentially inappropriate, 29% were potentially avoidable—assessed with ACSCs. The three highest ranked of these were COPD/pneumonia (26.3%), urinary tract infections (22.5%), and congestive heart failure (13.4%).

As might be expected, potentially inappropriate ED visits received significantly lower triage classifications; and visits initiated by ambulance calls were rarely potentially inappropriate. When compared to admissions by ambulance, patients who walked in were more likely to be there inappropriately (OR 3.42, 95%CI 1.79-6.55) as were those referred by their GPs (OR 2.13, 95%CI 1.16-3.90). The fact that referrals by GPs had higher odds of being inappropriate than ambulance calls seems counterintuitive: GPs are in a position to adequately assess and triage a situation in a nursing home. We assume that inappropriate GP referrals were more likely in situations where GPs had to rely on the assessments of nursing home care staff without being able to examine the patients personally. On the other hand, potentially avoidable visits were more often admitted as ambulance arrivals than as walk-in patients (OR for walk-ins 0.46, 95%CI 0.27-0.77), but more often still by GPs (OR 1.40, 95%CI 1.00-1.94).

As might also be expected, patients delivered by ambulance for potentially avoidable issues also had higher odds of being hospitalized than their walk-in counterparts. Compared to appropriate ED visits, potentially inappropriate ones led to significantly lower resource use; however, we found no overall difference between potentially avoidable and non-avoidable ED visits regarding ED resource use.

Compared to international findings, a 6% incidence of potentially inappropriate visits is rather low. E.g., a similar French study indicated 18.1% [15], an Australian one 13% [17], and a US one 36% [32]. Part of this comparative lowness might result from our use of an explicit evaluation protocol. In our sample, e.g., procedures that cannot be performed in nursing homes, e.g., radiological examinations, adjustment of indwelling urethral catheters, etc., accounted for more than 10% of all nursing home residents’ ED visits. Although visits for these procedures were not rated as potentially inappropriate, some might have been manageable in ambulatory care settings. As we did not have the information available to confirm such points, we treated them as appropriate. Similarly, some suspected fractures are manageable by GPs (during office hours) without reducing the resident’s quality of care. However, since in Switzerland not all GPs have the capacity for radiological examinations, only suspected fractures that did not require radiological examination were considered potentially inappropriate. Thus, while the low incidence of potentially inappropriate visits might accurately reflect the situation in nursing homes, it is also possible that we simply underestimated the number due to a lack of information.

One important point is that an appropriate visit could also be avoidable: whereas a visit’s appropriacy depends on the patient’s condition at the time of the visit, avoidability depends on possibilities for early detection and treatment of deteriorating symptoms. For example, following a diagnosis for congestive heart failure, appropriate medication and monitoring will normally prevent acute exacerbations that require ED visits. Compared to rates of avoidable visits in literature, which vary from 4% to 55% [8], our 29% incidence of avoidable visits was moderate.

Our distribution of ACSC frequencies was similar to those reported elsewhere. Our most common ACSC, pneumonia/COPD, accounted for 26.2% of our sample’s potentially avoidable visits. In comparison, this classification accounted for 30.1% of Gruneir and colleagues’ potentially avoidable visits [5] and 30.2% of Hsieh and colleagues’ [10]; where urinary tract infections accounted for 22.5%, Gruneir et al. found 20.3% [5] and Hsieh et al. 25.2% [10]. And where congestive heart failure accounted for 13.4% of our potentially avoidable visits, it represented 15.9% of Gruneir et al’s [5]. Similarly, our finding that ACSCs were related to higher hospitalization rates confirms the findings of several other studies [5, 10, 33]. In such cases, Gruneir et al. [5] suspected that the ACSCs’ deterioration had made hospitalization necessary.

Regardless of visits’ appropriateness or the presence of ACSCs, we found high levels of multimorbidity: our sample’s median CCI score was 6. This finding corresponds with other data from Switzerland, where nursing home residents have higher mean levels of multimorbidity than their community-dwelling peers [4]. Roughly 85.5% have at least two separate diagnoses; 22.8% have five or more chronic diagnoses [4]. Such high levels of comorbidity contribute to polypharmacy, which leads to increases in healthcare utilization, hospitalization rates [34] and ED overcrowding. Therefore, our group’s high comorbidity prevalence underscores the importance of interventions that address nursing home residents in holistic and coordinated ways, with due attention to overall chronic care management [35].

While the ED resource consumption of our sample’s potentially inappropriately admitted patients was significantly lower than for those who were appropriately admitted, they still used a total of 49’295 tax points (corresponding to approx. the same amount of US$) over the 3-year period assessed. Given the extra costs and limitations of emergency services entail regarding individualized care, every inappropriately-admitted patient could almost certainly have been treated both more cost-effectively and more safely elsewhere. Further, compared to the appropriately admitted group, potentially inappropriately admitted patients consumed smaller mean amounts of nurse work, laboratory and radiology resources; however, mean physician-patient time and medical reporting time did not differ between the two groups. I.e., inappropriate ED visits use resources that would clearly be better invested elsewhere.

Reviewing the reports for potentially inappropriate visits, we found that patients were often transferred for check-ups or examinations, while no acute event was listed in the patient’s history. Possible causes for such omissions include the lack of timely access to the patient’s treating physician [36] or a geriatric opinion [15], a failure to accurately assess the situation’s acuity, or simply the residents’ or their relatives’ preference to visit the ED. In a French study, Rolland et al. corroborated this possibility, observing that residents with access to both specialist medical advice and a mobile emergency medical unit were less likely to experience potentially inappropriate ED transfers [12].

Similarly, outreach teams or local interdisciplinary teams have been shown to reduce ED visits [37]. Given that 9% of our population’s visits were walk-in arrivals, another way of decreasing such ED visits might be to provide emergency walk-in doctors’ offices. Other patients in our sample whose visits were rated as potentially inappropriate were recommended admission for critical conditions, but opted to be transferred back to their nursing homes, as they wanted to abstain from invasive interventions. This indicates that some of the admissions might have been avoided by Advanced Care Planning (ACP), i.e., the assessment of and continuous conversation about residents’ and their families’ wishes for treatment in acute situations. ACP has been shown to be effective in reducing hospital admissions [38].

Unlike nursing home residents whose visits are potentially inappropriate, those transferred to the ED for potentially avoidable causes, especially poorly-managed ACSCs, incur the same mean resource costs as other appropriate visits (e.g., physician work, radiology resources), and more of others (physician-patient time, admin and medical report time, nurse work, laboratory resources). Therefore, over the 3-year study period, ACSC patients’ overall resource use was much higher than for inappropriate admissions. This corroborates the need to improve chronic care management in nursing homes as a strategy to reduce avoidable ED referrals. The current gap in management could be bridged via improved access to primary care providers—either GPs or nurse practitioners; or, within each nursing home, a structured medical system could be implemented to provide both on-site expertise and quick access to it.

Nursing home residents with regular opportunities to engage with their primary care providers are less likely to have ED visits [39]. To test and, if possible, optimize this connection, an ongoing US initiative is placing geriatric-specialized nurse practitioners in nursing homes. By supporting early recognition and initiating interventions to stabilize worsening conditions, these practitioners have reduced all-cause hospitalizations by up to 30% [40]. Especially in nursing homes with a majority of low-skilled care workers, this initiative shows the value of geriatric expertise (or other forms of geriatric-specialized services), strong advance care planning, and palliative care [41].

The present investigation is limited by several factors. Firstly, no classification system for potentially inappropriate hospitalizations has yet been used broadly to allow for international comparison. For the sample described, we distinguished appropriate from potentially inppropriate visits based on the development of a protocol by a clinical review panel in Australia [17]. Other appropriateness evaluation protocols have been used elsewhere [15, 16], which might increase the heterogeneity between our and earlier researchers’ results; and our use of a single instrument may bias the current analysis. However, when we were starting our data analysis, Finn and colleagues’ instrument was designed specifically for ED visits (as opposed to hospitalizations) [17], so we deemed it the most suitable yet published. Within the protocol's recommended procedure, one critical step was to determine whether it would have been possible to perform an observation or procedure in the nursing home (Supplement 1). Although we predefined scenarios for such observations or procedures, their validity remains unsure: we could not obtain data about the relevant resources’ availability at the nursing homes in question, e.g., their access to primary care and possibilities for risk assessments. To reduce the risk of incorrect classification, we had two trained raters classify the visits independently.

As for relying on the presence of ACSCs as avoidability indicators, the raters were evaluating only diagnoses. I.e., while other factors—e.g., nursing homes’ in-house treatment options, the availability of appropriately-qualified staff, the presence of a GP or how closely their policies adhered to their residents’ wishes—may have been influential to other decisions, the raters did not consider them. Furthermore, we had no access to ICD-10 diagnoses, which are not used in EDs. Instead, we had text versions of the involved physicians’ diagnoses. For future researchers, this limits the comparability of this study’s diagnoses to those obtained elsewhere.

An additional limitation was that we had access only to ED data: we had no information about nursing home staffs’ reasons for initiating transfers, the challenges or needs affecting them or the resources accessible to them at the time of the transfer. Therefore, we can give no definitive suggestions to improve the link between nursing homes and the medical care centers in the ED’s catchment area. Moreover, in the ED, data on our sample patients’ end-of-life planning were not systematically obtained. This hindered our assessment of problems regarding ACP or do-not-hospitalize orders. The study also has a risk of selection bias due to exclusion of patients who objected to the use of their data in health research; and as this is a retrospective chart review, it is prone to documentation error. Finally, some categories of the predictor variables contain relatively few cases, leading to large confidence intervals for certain variables (e.g., ‘No connective tissue disease’).

## Conclusion

Potentially inappropriate visits from nursing homes to the study center’s ED were relatively rare but may have been underestimated. Lack of urgency and re-transfers of patients requiring palliative care were the most common reasons for classing referrals as inadequate. Preventive strategies focusing on high-quality ambulatory care might further reduce potentially inappropriate emergency admissions. To ensure comparability between studies, future research should focus on standardization of appropriateness evaluation protocols. From the ED’s perspective, high resource use for visits due to ACSCs underscores the need to address chronic disease management in nursing homes. And finally, to enable the development and exploration of interventions that address the factors driving potentially inappropriate and/or avoidable ED visits, further investigation is required regarding methods of identifying such visits.

## Supplementary Information

**Additional file 1.**

## Data Availability

The datasets used and/or analyzed during the current study are available from the corresponding author upon reasonable request.

## Abbreviations

ACP: Advance care planning
ACSC: Ambulatory-care sensitive conditions
CCI: Charlson Comorbidity Index
CI: Confidence Interval
COPD: Chronic obstructive pulmonary disease
CT: Computer Tomography
ED: Emergency Department
GP: General practitioner
ICU: Intensive care unit
IQR: Interquartile range
LOS: Length of stay
Med: Median
TP: Tax point
