# Supplementary material for: Inappropriate and potentially avoidable emergency department visits of Swiss nursing home residents and their resource use: a retrospective chart-review
Source: BMC Geriatr. 2022 Aug 11;22:659. doi: 10.1186/s12877-022-03308-9 (PMC9367060; doi:10.1186/s12877-022-03308-9)
Supplement: Supplementary file 1 — Additional file 1. [file 12877_2022_3308_MOESM1_ESM.pptx]

## Slide 1
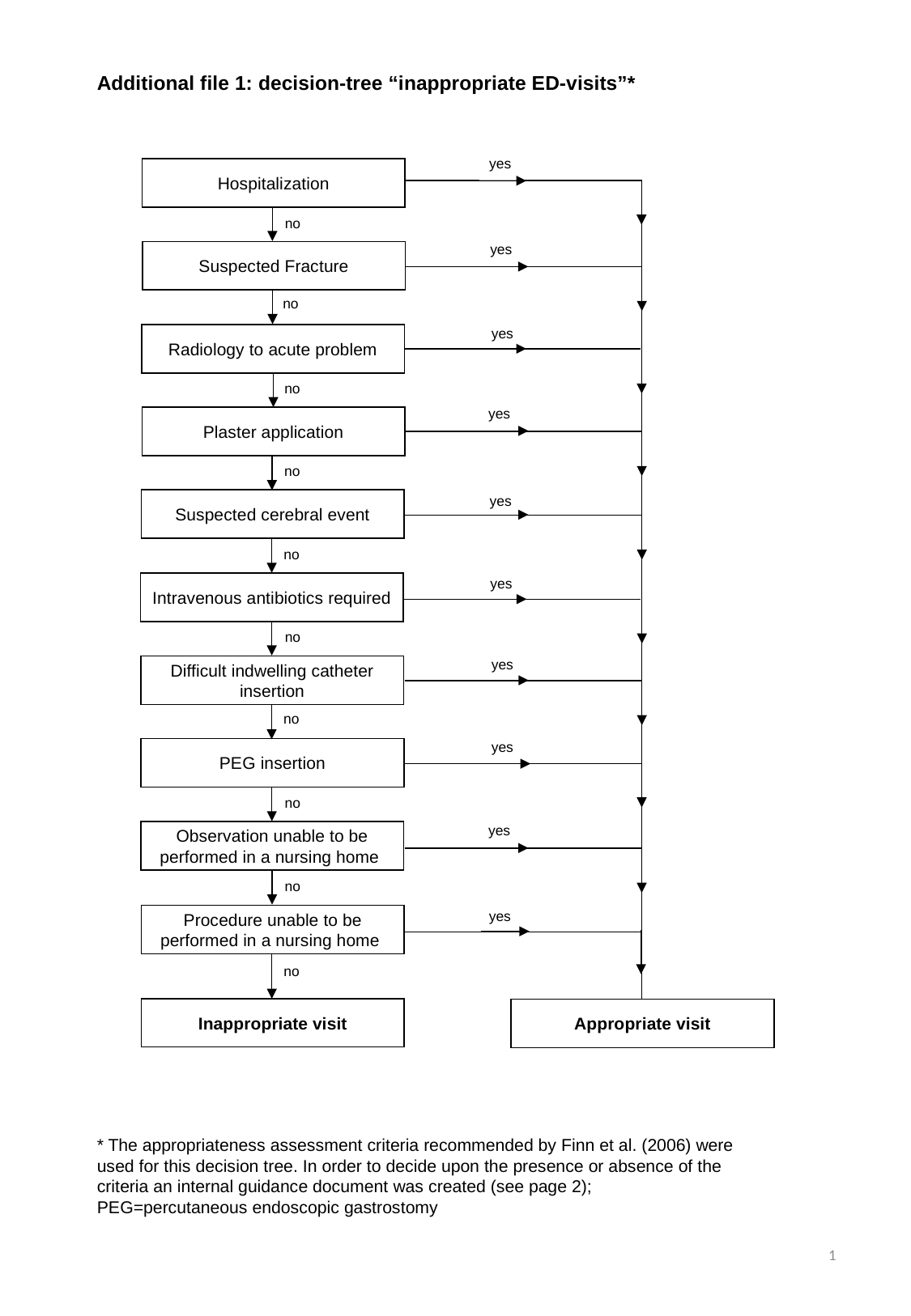

Additional file 1: decision-tree “inappropriate ED-visits”*
yes
Hospitalization
no
yes
Suspected Fracture
no
yes
Radiology to acute problem
no
yes
Plaster application
no
yes
Suspected cerebral event
no
yes
Intravenous antibiotics required
no
yes
Difficult indwelling catheter insertion
no
yes
PEG insertion
no
yes
Observation unable to be performed in a nursing home
no
yes
Procedure unable to be performed in a nursing home
no
Inappropriate visit
Appropriate visit
* The appropriateness assessment criteria recommended by Finn et al. (2006) were used for this decision tree. In order to decide upon the presence or absence of the criteria an internal guidance document was created (see page 2);
PEG=percutaneous endoscopic gastrostomy
1

## Slide 2
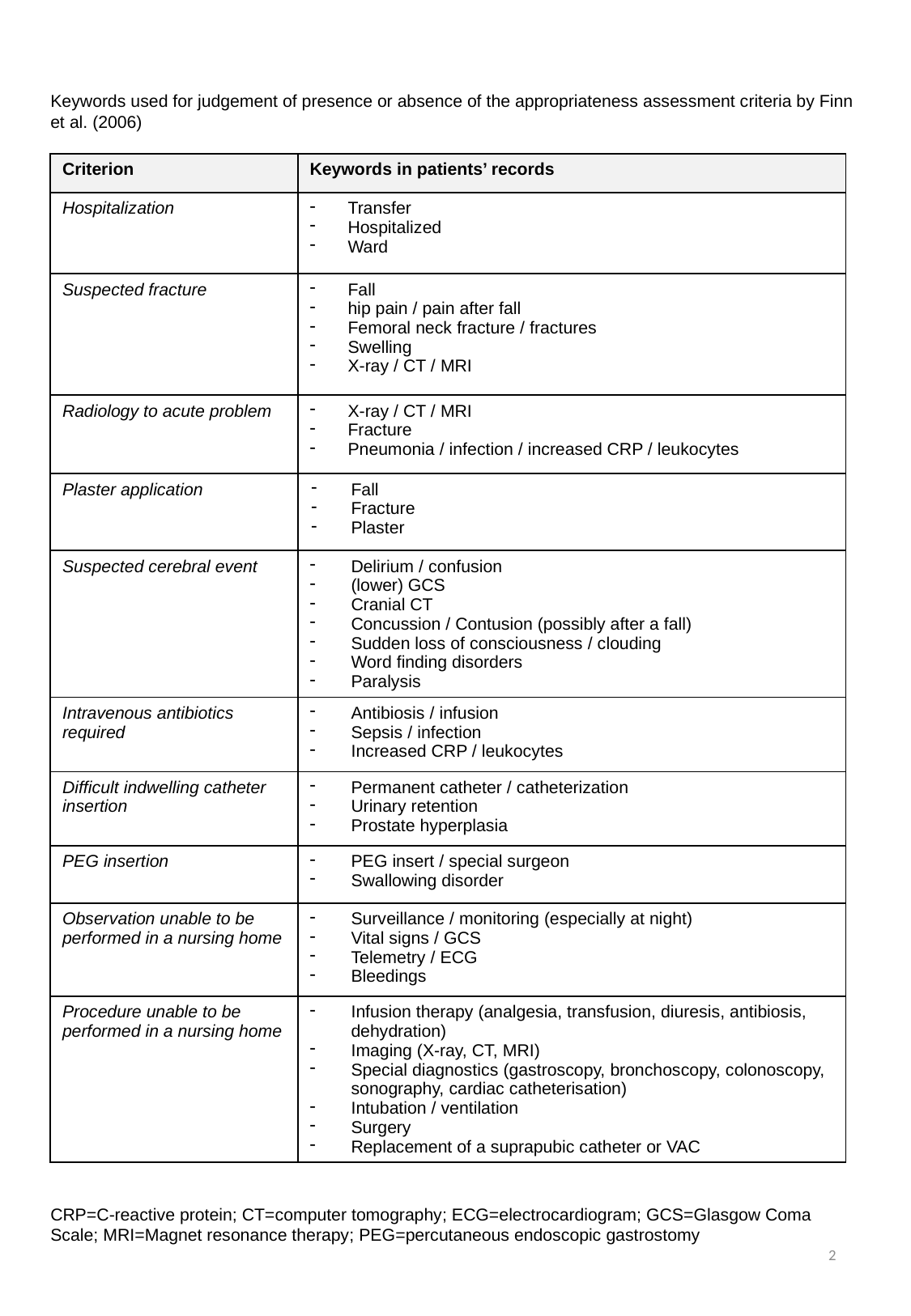

Keywords used for judgement of presence or absence of the appropriateness assessment criteria by Finn et al. (2006)
| Criterion | Keywords in patients’ records |
| --- | --- |
| Hospitalization | Transfer Hospitalized Ward |
| Suspected fracture | Fall hip pain / pain after fall Femoral neck fracture / fractures Swelling X-ray / CT / MRI |
| Radiology to acute problem | X-ray / CT / MRI Fracture Pneumonia / infection / increased CRP / leukocytes |
| Plaster application | Fall Fracture Plaster |
| Suspected cerebral event | Delirium / confusion (lower) GCS Cranial CT Concussion / Contusion (possibly after a fall) Sudden loss of consciousness / clouding Word finding disorders Paralysis |
| Intravenous antibiotics required | Antibiosis / infusion Sepsis / infection Increased CRP / leukocytes |
| Difficult indwelling catheter insertion | Permanent catheter / catheterization Urinary retention Prostate hyperplasia |
| PEG insertion | PEG insert / special surgeon Swallowing disorder |
| Observation unable to be performed in a nursing home | Surveillance / monitoring (especially at night) Vital signs / GCS Telemetry / ECG Bleedings |
| Procedure unable to be performed in a nursing home | Infusion therapy (analgesia, transfusion, diuresis, antibiosis, dehydration) Imaging (X-ray, CT, MRI) Special diagnostics (gastroscopy, bronchoscopy, colonoscopy, sonography, cardiac catheterisation) Intubation / ventilation Surgery Replacement of a suprapubic catheter or VAC |
CRP=C-reactive protein; CT=computer tomography; ECG=electrocardiogram; GCS=Glasgow Coma Scale; MRI=Magnet resonance therapy; PEG=percutaneous endoscopic gastrostomy
2
